# Perceptual Grouping During Binocular Rivalry in Mild Glaucoma

**DOI:** 10.3389/fnagi.2022.833150

**Published:** 2022-05-25

**Authors:** Galia Issashar Leibovitzh, Graham E. Trope, Yvonne M. Buys, Luminita Tarita-Nistor

**Affiliations:** ^1^Krembil Research Institute, Donald K. Johnson Eye Institute, University Health Network, Toronto, ON, Canada; ^2^Department of Ophthalmology and Vision Sciences, Toronto Western Hospital, Toronto, ON, Canada

**Keywords:** glaucoma, binocular rivalry, grouping, visual processing, neural connectivity, optic neuropathy

## Abstract

**Purpose:**

This study tested perceptual grouping during binocular rivalry to probe the strength of neural connectivity of the visual cortex involved in early visual processing in patients with mild glaucoma.

**Methods:**

Seventeen patients with mild glaucoma with no significant visual field defects and 14 healthy controls participated. Rivalry stimuli were 1.8°-diameter discs, containing horizontal or vertical sine-wave gratings, viewed dichoptically. To test the grouping, two spatially separated identical stimuli were presented eccentrically to the same or different eyes and to the same or different hemifields. The outcome measures were the time of exclusive dominance of the grouped percept (i.e., percept with synchronized orientations), the rivalry rate, and the epochs of exclusive dominance.

**Results:**

For both groups, the grouping occurred primarily for the matching orientations in the same eye/same hemifield (MO SE/SH) and for the matching orientations in the same eye/different hemifield (MO SE/DH) conditions. Time dominance of the grouped percept of the glaucoma group was similar to that of the control group in all conditions. The rivalry rates in the MO SE/SH and MO SE/DH conditions were significantly larger in the control group than in the glaucoma group. The epochs of exclusive dominance of the grouped percept in the MO SE/SH condition were a median of 48-ms longer for the control group, but a median of 116-ms shorter for the glaucoma group when compared to those in the MO SE/DH condition.

**Conclusion:**

Patients with mild glaucoma show clear impairments in binocular rivalry while evidence for deficits in perceptual grouping could be inferred only indirectly. If these deficits truly exist, they may have implications for higher levels of visual processing, such as object recognition and scene segmentation, but these predictions remain to be tested in future studies.

## Introduction

Glaucoma is a progressive eye disease that affects people over 40 years of age and is the leading cause of global irreversible blindness (Quigley and Broman, 2006; Varma et al., 2011; Tham et al., 2014; Torabi et al., 2021; Zhang et al., 2021). As the population continues to age and the average life expectancy increases, it is expected that the number of people affected by this debilitating disease to increase worldwide from an estimated 76 million in 2020 to 112 million in 2040 (Tham et al., 2014). Patients with glaucoma have impairments with aspects of visual processing and functional vision (Silverman et al., 1990; Ramulu, 2009; Black et al., 2011; Tarita-Nistor et al., 2014, 2019; Brin et al., 2019) and therefore early detection is important for efficient treatment and disease management.

Glaucoma is considered an optic neuropathy because of the damage and death of the retinal ganglion cells (RGCs) (Quigley, 1999); however, this loss must be substantial (i.e., 20–40% RGC loss) before changes in the vision are detected with standard automated perimetry tests (Tai, 2018). Glaucoma is also considered a neurological disease because it affects neural structures not only in the primary visual pathways (Gupta et al., 2006; Garaci et al., 2009; Hernowo et al., 2011; Zhang et al., 2012) and visual association areas but also in the other parts of the brain, including the corpus callosum—the neural bundle that connects the two brain hemispheres and is involved in the inter-hemispheric transfer (Williams et al., 2013; Yu et al., 2013; Frezzotti et al., 2014; Boucard et al., 2016; Jiang et al., 2017; Nucci et al., 2020). While some degeneration in the primary visual pathways can be explained by anterograde trans-synaptic axonal degeneration, changes in the more distal neural structures suggest separate degenerative mechanisms. Indeed, it has been proposed that glaucoma shares common neurodegenerative as well as neuroinflammatory mechanisms with classical neurodegenerative pathologies, such as Alzheimer’s and Parkinson’s diseases (Ramirez et al., 2017).

The functional integrity of some brain structures can be probed with behavioral tests. For example, we have recently shown that a test based on binocular rivalry detects the early deficits in the inter-hemispheric transfer—suggestive of callosal dysfunction—in patients with mild glaucoma (Tarita-Nistor et al., 2020). Binocular rivalry is a phenomenon that has been used extensively to examine the dynamics of the visual system in healthy and diseased (Miller et al., 2003; Knapen et al., 2007; Grossberg et al., 2008; Robertson et al., 2013). When two different images are presented dichoptically (e.g., one eye sees the horizontal gratings and the other eye sees the vertical gratings), the visual system cannot integrate them into one stable percept; rather, the two images compete for visual awareness, such as one is dominant and the other is suppressed, in an order that is reversed—moments later—in a wave-like fashion and in a continuous cycle. Binocular rivalry can be tested in the same hemifield (i.e., left or right) and in both hemifields (requiring inter-hemispheric transfer of visual information) depending on the location of the rivalrous targets. We found that—compared to the healthy controls—the patients with mild glaucoma have lower binocular rivalry rates (Tarita-Nistor et al., 2020) and a disruption in the dominance wave propagation (Tarita-Nistor et al., 2019) when binocular rivalry involves both hemifields. The former finding has been replicated independently (João et al., 2021) bringing additional evidence for the impairment of the inter-hemispheric processing during the binocular rivalry in mild glaucoma.

**FIGURE 1:**
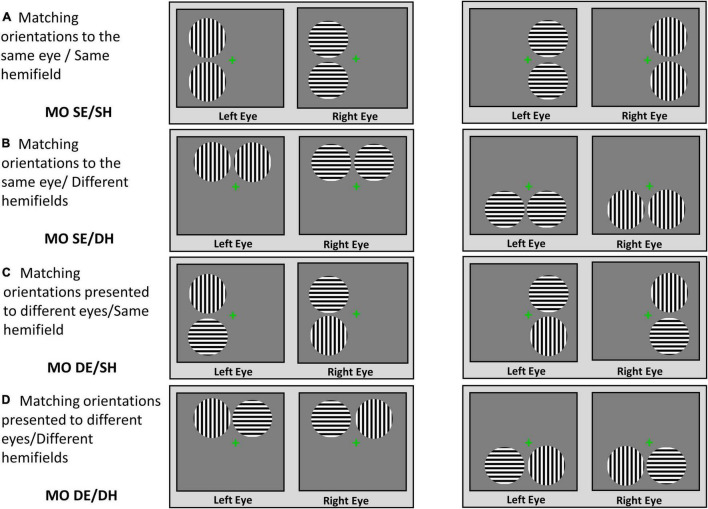
Stimuli and viewing conditions. Perceptual grouping during the binocular rivalry was tested in the following viewing conditions: **(A)** MO SE/SH; **(B)** MO SE/DH; **(C)** MO DE/SH; **(D)** MO DE/DH.

During the binocular rivalry, spatially separated stimuli with similar features, such as orientation tend to group together; that is, they are perceived at the same time. Grouping may be mediated by lateral connections of the cortical hypercolumns in the visual cortex (Alais and Blake, 1999). In controls, there is a slightly stronger perceptual grouping for targets presented in the same hemifield than in both hemifields in conditions involving eye-based dominance of an image (i.e., one eye sees the two identical images, spatially separated, that are presented in the same or in both hemifields, while the other eye sees the other two identical images presented in similar arrangements, as shown in Figures 1A,B). These findings indicate a stronger connectivity between the adjacent rivalry zones within than that between hemifields (Stuit et al., 2011, 2014).

Perceptual grouping during a binocular rivalry paradigm can be used to probe the strength of neural connectivity of the visual cortex involved in the early visual processing. A weakening of the connectivity in the early visual cortex may have implications for higher levels of visual processing, such as object recognition and scene segmentation, and may have important consequences for functional vision; for example, finding an object in a crowded environment and in recognizing pedestrians while driving. Therefore, the purpose of this study was to examine the perceptual grouping during binocular rivalry in patients with mild glaucoma who otherwise have no significant visual deficits on standard clinical measures. We hypothesized that the patients with mild glaucoma may show impairments in grouping during binocular rivalry possibly due to early neurodegenerative processes.

## Materials and Methods

### Participants

The participants included patients with mild-open angle glaucoma recruited from referrals to the eye clinic at the Toronto Western Hospital, University Health Network, Toronto, Canada, and the healthy controls recruited from staff, collaborators, and their spouses. The study was conducted during the COVID-19 pandemic, when restrictions implemented by our institution prevented us from expanding the recruitment of healthy controls to other volunteers from the general population. Testing of all participants was performed in the ocular motor laboratory at the same hospital. Ethics approval was obtained from the University Health Network’s Research Ethics Board and the research was conducted in accordance with the tenets of the declaration of Helsinki. Written informed consent was obtained from all participants after the study was explained to them in detail.

For the patients, a diagnosis of mild glaucoma was made by an experienced glaucoma specialist (the author GET) and was based on changes consistent with the diagnosis of mild glaucoma shown by consecutive optical coherence tomography (OCT), visual field test and clinical examinations of the status of the optic disc and intraocular pressure (IOP). The grading scale used was Hodapp–Parrish Anderson and corresponded to stage 0–1 [i.e., mean deviation (MD) from 0 to –6 dB]. The patients were included in the study if they had a confirmed diagnosis of mild bilateral open-angle glaucoma and no other important comorbid ocular pathologies, no significant functional or structural asymmetries between the two eyes, and no substantial monocular or binocular functional deficits. All patients included in the study were treated by the glaucoma specialist and their IOP was below 21 mm Hg. Controls were included if they had healthy vision with no known significant ocular pathologies or functional and structural deficits in the 2 eyes. The control participants confirmed verbally that they had had an ophthalmic examination within 2 years, there were no pathological findings or suspicion of any eye diseases, and their habitual correction was updated.

The patients with more advanced stages of glaucoma and/or a history of major retinal or corneal eye surgeries were excluded from this study. The patients and healthy controls with cognitive impairment, a history of neurological disease, diabetes, or other significant ocular diseases except for symmetric mild cataracts were also excluded. In addition, the participants who met all the inclusion criteria but nevertheless were not able to fuse the fixation crosses during the rivalry experiment were excluded. The demographic and clinical characteristics are shown in Table 1 in “Results” section.

**TABLE 1:** Demographic and clinical characteristics for glaucoma and control group, presented as the mean ± standard deviation.

	Glaucoma	Control	*P*
N [M/F]	17 [8/9]	14 [7/7]	–
Age (years)	61 ± 12	53 ± 11	0.06
Stereo acuity (s)	64 ± 103	74 ± 138	0.82
**Visual acuity 96% contrast (logMAR)**			
Binocular	–0.07 ± 0.05	–0.08 ± 0.09	0.56
Right eye	–0.03 ± 0.06	–0.05 ± 0.08	0.23
Left eye	–0.04 ± 0.05	–0.05 ± 0.09	0.47
**Visual acuity 25% contrast (logMAR)**			
Binocular	0.06 ± 0.10	0.04 ± 0.10	0.62
Right eye	0.11 ± 0.10	0.07 ± 0.13	0.30
Left eye	0.10 ± 0.10	0.10 ± 0.15	0.92
**Visual field mean deviation (dB)**			
Right eye	0.23 ± 1.31	–	–
Left eye	–0.47 ± 1.43	–	–
**Retinal nerve fiber layer (μm)**			
Right eye	83 ± 12	–	–
Left eye	81 ± 12	–	–
**Average cup-to-disc ratio**			
Right eye	0.62 ± 0.15	–	–
Left eye	0.65 ± 0.14	–	–
**Vertical cup-to-disc ratio**			
Right eye	0.60 ± 0.16	–	–
Left eye	0.64 ± 0.14	–	–

### Apparatus and Stimuli

For both groups, visual acuity was measured at a 6-m distance with a computerized version of the Early Treatment Diabetic Retinopathy Study (ETDRS) chart (single line) using the Accommodata Stimuli System, Version 3.5 (Haag–Streit, Mason, OH). Monocular and binocular visual acuities were obtained at high (95%) and low (25%) contrast with the participants’ habitual glasses. A letter-by-letter scoring system was used and the thresholds were reported in logMAR units. Stereoacuity was measured with the Random Dot Stereoacuity Test (Good–Lite Company, Elgin, IL).

For the glaucoma group, only clinical tests were conducted as part of the standard of care during their regular appointment with their glaucoma specialist. These tests included monocular visual field sensitivity assessed for each eye using the Humphrey Field Analyzer (Humphrey Field Analyzer; model HFA-II 750; Carl Zeiss Meditec, Dublin, CA) utilizing the monocular 24–2 Swedish Interactive Threshold Algorithm–Standard. The MD values were obtained only from reliable tests (i.e., with less than 20% fixation losses and less than 33% false positive and false negative responses). In addition, peripapillary retinal nerve fiber layer (RNFL) thickness, average cup-to-disc ratio, and vertical cup-to-disc ratio were recorded with spectral domain OCT (model Cirrus; Carl Zeiss Meditec, Dublin, CA), using a 200 × 200 optic disc cube protocol scan. Visual field and OCT measures were not obtained from the healthy controls due to the safety concerns from COVID-19 exposure and restricted access to the OCT and visual field machines to patients only.

Psychophysical measures recorded for the perceptual grouping during the binocular rivalry task were obtained for both groups. The rivalry stimuli were generated with VPixx, a graphics and psychophysics software (VPixx Technologies, Inc., Montreal, QC), and presented on an iMac computer screen with a resolution of 1,680 × 1,050 pixels. These stimuli were viewed through a double-mirror stereoscope placed in front of the computer. To ensure that each eye saw a different image, the stimulus area on the monitor was surrounded by a black mask and a vertical black divider was placed perpendicularly between the stereoscope and the monitor. A 0.5° green fixation cross was presented centrally in all conditions; eccentricity was measured from the middle of this fixation to the stimulus’ center and was constant in all conditions at ± 1° in the horizontal and the vertical directions, respectively (i.e., 1.41° on a diagonal from center of the stimulus to the center of the cross). The rivalry stimuli were 1.8°-diameter discs, containing horizontal or vertical sine-wave gratings with a spatial frequency of 4 cpd. To test grouping during binocular rivalry, two spatially separated adjacent rivalry stimuli (with a separation gap of 0.2°) were shown, such as identical targets (i.e., two discs with horizontal gratings or two discs with vertical gratings), were presented to the same or different eye, and to the same or different hemifield. That is, the following viewing conditions were tested: (1) Matching orientations in the same eye/same hemifield (MO SE/SH; Figure 1A), in which the identical rivalry targets were presented to the same eye (i.e., both horizontal to the left eye and both vertical to the right eye, or *vice versa*) and to the same hemifield (i.e., left hemifield or right hemifield); (2) matching orientations in the same eye/different hemifields (MO SE/DH; Figure 1B), in which the identical rivalry targets were presented to the same eye but in different hemifields, above or below the fixation cross; (3) matching orientations in different eyes/same hemifield (MO DE/SH; Figure 1C) in which the identical rivalry targets were presented to different eyes (i.e., one horizontal to the left eye in the upper position and to the right eye in the lower position, and one vertical to the left eye in the lower position and to the right eye in the lower position, or *vice versa*) and to the same hemifield, and (4) matching orientations in different eyes/different hemifield (MO DE/DH; Figure 1D), in which the identical rivalry targets were presented to different eyes and to different hemifields, above or below the fixation cross. In each condition, the sine-wave grating’s orientation of the stimuli (i.e., horizontal or vertical) and location (i.e., left or right; above or below) were counterbalanced, creating a total of eight conditions that were fully randomized. In addition, a central-control condition in which the traditional single rivalry stimuli were presented centrally was included. These single rivalry targets consisted of the same 1.8°-diameter discs, containing sine-wave gratings with a spatial frequency of 4 cpd, one disc with horizontal orientation presented to one eye and one disc with vertical orientation presented to the other eye. The 0.5° fixation cross was maintained for this condition too. The central-control condition was useful for the following two reasons: (1) it helped in explaining the binocular rivalry paradigm and ensured that the participants understood it, and (2) it verified the replicability of our previous reports (Tarita-Nistor et al., 2020).

A button-response box with two active buttons connected to a personal computer and in-house software written in Visual Basic (Microsoft, Albuquerque, NM, US) were used to record the total time dominance of each percept as well as the rivalry rate (i.e., the number of perceptual switches per minute) for each condition. The two response buttons had tactile cues attached to them to indicate which button corresponded to each stimulus orientation.

### Procedure

**FIGURE 2:**
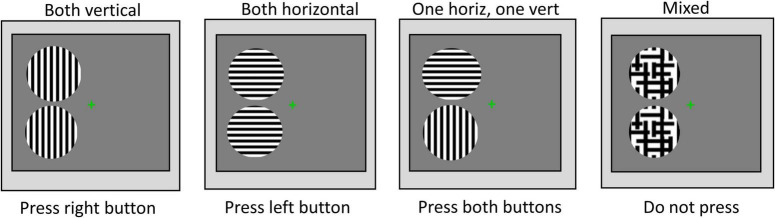
Instructions for percept dominance responses. Examples of the possible rivalry percepts and instructions on how to press buttons of the response box.

Visual acuity, stereoacuity, and the psychophysical measures related to the perceptual grouping task were undertaken in a single 1-h-long laboratory session. After the experiment was explained and written informed consent obtained, the perceptual grouping during the binocular rivalry test was started. The apparatus was placed on a table that could be adjusted, such as the center of the screen was at the eye level for each participant. A chin rest was also attached to this table to stabilize the head while participants were seated on an adjustable chair. The participants were instructed to look through the double-mirror stereoscope and report if they saw one green cross or two (i.e., the fixation stimuli that were projected on the computer screen). If the participants were able to see a single fixation cross, the experiment proceeded further. The participants were told to keep their gaze fixed on the cross and were instructed to press the buttons on the button-response box as follows: Press the right button if both discs were perceived to have vertical gratings, press the left button if both discs were perceived to have horizontal gratings, or both buttons (i.e., press the right and the left buttons simultaneously) if one disc was perceived to have horizontal gratings while the other disc was perceived to have vertical gratings. If the percept was a mixture of horizontal and vertical gratings in any of the discs (e.g., piecemeal or superimposed percepts), the participants were instructed not to press any buttons. The participants were shown a printout of possible percepts (see Figure 2), but it was explained to them that the position of the two discs also could be to the right, above, and below to the fixation cross, for which the instructions on how to press the buttons were the same. The total time the left button, the right button, and both buttons together were pressed, respectively, as well as the time for no button press were recorded for each test condition. The total time for the mixed percept included the piecemeal and superimposed percepts, but given the small stimulus size, the time dominance of mixed percept was expected to be low (Blake et al., 1992).

After all the adjustments were made, the testing was performed in a darkened room, where all light sources—except for the computer monitor showing the stimuli—were eliminated. A practice trial was first run using the central-control condition. Then, the eight conditions for testing the perceptual grouping during the binocular rivalry were run in a random order. The central-control condition was also included in this block. Each condition was 1-min long and a short break was allowed after each condition. The participants were repeatedly reminded to keep their eyes fixated on the central green cross, but to pay attention to the eccentric rivalrous stimuli.

After the binocular rivalry task was completed, the monocular and binocular visual acuities at high and low contrast, as well as the stereoacuity thresholds were measured for all participants in the laboratory. The OCT scans and visual field tests were taken as a part of the follow-up standard of care in the same day or up to 6 months prior to the experiment.

### Data Analysis

As shown in Figure 1 and described above, there were two trials for each experimental condition (i.e., MO SE/SH, MO SE/DH, MO DE/SH, and MO DE/DH) and the outcome measures were averaged for each condition. The main outcome measures were as follows: (1) The time of exclusive dominance of the percept with synchronized orientations (i.e., both horizontal or both vertical, cumulative within a trial, indicating grouped percept), with unsynchronized orientations (i.e., one horizontal and one vertical, indicating ungrouped percept), as well as mixed percept, (2) the rivalry rate reported as the number of perceptual switches per minute, and (3) the epochs of exclusive dominance of the grouped percept, defined as time of exclusive dominance of this percept divided by the rivalry rate, computed only for the MO SE/SH and the MO SE/DH conditions where grouping occurred.

Parametric tests, such as paired-samples *t*-test, independent-samples *t*-test, and correlations, were used. For the most part, the data were analyzed with mixed factorial analysis of variance (ANOVA). In cases of multiple comparisons, the familywise error rate was controlled with the Bonferroni approach. When violations of sphericity assumption were detected, the ANOVA effects were adjusted using the Greenhouse–Geisser correction. Alpha level was set at 0.05 for all tests.

## Results

### Participants

Seventeen patients (mean age, 61 ± 12 years; 8M/9F) with mild open-angle glaucoma and 14 controls (mean age, 53 ± 11 years; 7M/7F) completed the study. Independent samples *t*-tests showed there was no significant difference in age, stereoacuity, or monocular and binocular visual acuity at high and low contrast between the two groups. Means and standard deviations of these measures along with the *p*-values are shown in Table 1. In addition, interocular differences (i.e., left eye vs. right eye measures) were evaluated with paired-samples *t*-tests separately for each group. For the control group, no interocular differences in visual acuity at high and low contrast were found. Likewise, the glaucoma group showed no interocular differences in visual acuity at high and low contrast, as well as in average cup-to-disc ratio and vertical cup-to-disc ratio. The small asymmetries in visual field’s MD and in RNFL thickness measures were observed between the left and the right eyes — although statistically significant — they were of no clinical relevance (Waisbourd et al., 2015; Jiang et al., 2020), and no patient was identified as having asymmetric deficits by the glaucoma specialist. Means and standard deviations for the visual field’s MD and the structural measures derived from the OCT tests for the glaucoma group only are also shown in Table 1.

### Percept Dominance

Time dominance of the grouped, ungrouped, and mixed percept was assessed using separate 4 (condition: MO SE/SH, MO SE/DH, MO DE/SH, MO DE/DH) × 2 (group: glaucoma, control) mixed factorial ANOVAs. For the grouped percept, the main effect of condition was significant, [*F*(1.7, 48.2) = 232, *p* < 0.001, partial η^2^ = 0.89], but no significant interactions or group effects were detected. The pairwise comparisons showed that the time dominance for the grouped percept was longer in the MO SE/SH and in the MO SE/DH conditions than in the MO DE/SH and MO DE/DH conditions, all *p*-values were less than 0.001. There was no difference between MO SE/SH and MO SE/DH; and between MO DE/SH and MO DE/DH conditions. These results suggest that for both groups, a synchronized dominance (i.e., grouped percept) was longer when identical stimuli were presented to the same eye (i.e., both horizontal to one eye and both vertical to the other eye) than to different eyes irrespective of the hemifield.

**FIGURE 3:**
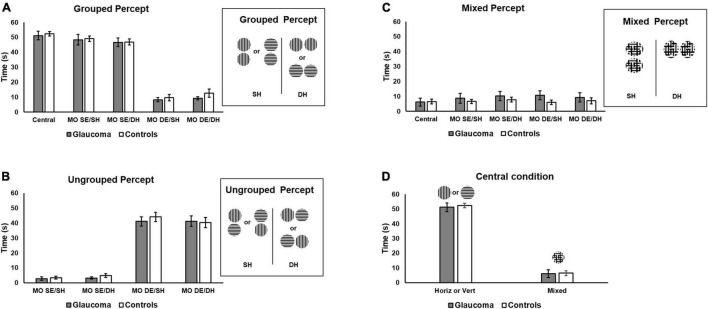
Time of exclusive percept dominance. Time dominance for the grouped, ungrouped, and mixed percept for the four rivalry conditions are shown in **(A–C)**, respectively. Grouped percept is the percept in which the orientations of the sinewave gratings are synchronized for both rivalry targets, as shown on the right of **(A)** for the same SH and different hemifields (DH). For the ungrouped percept, these orientations are not synchronized. **(D)** Includes the time of exclusive horizontal or vertical percept dominance for the central-control condition and that of the mixed percept for the same condition, respectively. Error bars are ± 1 SE.

Similar analysis was conducted for the ungrouped percept. The analysis revealed only that condition main effect was significant, [*F*(1.7, 49.9) = 215, *p* < 0.001, partial η^2^ = 0.88], but no interaction or group effects were found. The pairwise comparisons showed that the time dominance for the ungrouped percept was significantly shorter in the MO SE/SH and in the MO SE/DH conditions than in the MO DE/SH and MO DE/DH conditions, all *p*-values were less than 0.001, but the differences between MO SE/SH and MO SE/DH as well as MO SE/DH and MO DE/DH were not significant. These results are shown in Figure 3B. These findings imply that for both groups, unsynchronized dominance (i.e., ungrouped percept) was longer when identical stimuli were presented to different eyes (i.e., one horizontal and one vertical to one eye, and one vertical and one horizontal to the other eye) than to the same eye, irrespective of the hemifield.

Finally, a similar analysis was conducted for the mixed percept. The mixed factorial ANOVA revealed that the condition, group, or interaction effects were not significant, as shown in Figure 3C.

We further explored the overall differences in time dominance for the grouped and ungrouped percepts, using a 4 (condition: MO SE/SH, MO SE/DH, MO DE/SH, MO DE/DH) × 2 (percept: grouped, ungrouped) × 2 (group: glaucoma, control) mixed factorial ANOVA, for which only the percept main effect and percept interactions are reported. The percept main effect was significant, [*F*(1, 29) = 18.7, *p* < 0.001, partial η^2^ = 0.39]. Overall, the time dominance for the grouped percept was significantly higher than that for the ungrouped percept. In addition, only the percept × condition interaction effect was significant [*F*(3, 87) = 245, *p* < 0.001, partial η^2^ = 0.89]. This interaction effect can be best observed in Figures 3A,B. For both groups, the time dominance of the grouped percept was longer than that of the ungrouped percept in the MO SE/SH and MO SE/DH conditions, while the opposite was true for the MO DE/SH and MO DE/DH conditions (all *p*-values were less than 0.001). In addition, the time dominance of the grouped percept was significantly longer in the MO SE/SH and MO SE/DH than in the MO DE/SH and MO DE/DH conditions, while the opposite was true for the time dominance of the ungrouped percept (all *p*-values were less than 0.001).

For the central-control condition, an independent-samples *t*-test showed that the time of exclusive dominance (i.e., horizontal or vertical percept) did not differ for the two groups. Likewise, the time dominance for the mixed percept did not differ for the two groups, as shown in Figure 3D. We further compared the time of exclusive dominance of the central-control condition with those of the conditions conducive to grouped percept (MO SE/SH and MO SE/DH). A 3 (condition: central-control, MO SE/SH, MO SE/DH) × (group: glaucoma, control) mixed factorial ANOVA revealed that condition main effect was significant, [*F*(2, 56) = 3.87, *p* = 0.036, partial η^2^ = 0.12], but there were no group or interaction effects. Follow-up analysis showed a significant difference between the central-control and MO SE/DH conditions (*p* = 0.023), but this was true only when adjustments for multiple comparisons were performed with a less conservative test (i.e., Least significant difference (LSD) rather than Bonferroni). Also, when comparing the mixed percept of the central-control conditions with the other conditions, no significant results were found. Means and standard deviations of the time dominance the grouped, ungrouped, and mixed percept are presented in Table 2.

**TABLE 2:** Mean ± standard deviation of the time dominance for grouped, ungrouped, and mixed percept.

	Grouped (s)		Ungrouped (s)		Mixed (s)	
			
	Glaucoma	Control	Glaucoma	Control	Glaucoma	Control
MO SE/SH	48.4 ± 14	49.1 ± 7	2.8 ± 5	3.5 ± 4	8.8 ± 14	6.7 ± 5
MO SE/DH	46.6 ± 12	46.9 ± 8	3.3 ± 4	5.0 ± 5	10.2 ± 13	7.8 ± 6
MO DE/SH	8.3 ± 6	9.6 ± 8	41.1 ± 13	44 ± 11	10.7 ± 12	6.1 ± 6
MO DE/DH	9.3 ± 5	12.6 ± 10	41.3 ± 14	40.4 ± 13	9.3 ± 13	7.0 ± 8

*For the central-control condition, the average time of exclusive dominance (i.e., horizontal or vertical percept) was 51.2 ± 11 s for the glaucoma group and 52.4 ± 5 s for the control group, while the average mixed percept was 6 ± 11 s for the glaucoma group and 6.6 ± 6 s for the control group.*

### Rivalry Rate of the Grouped Percept

The rivalry rate was examined for the conditions conducive to grouped percept (i.e., MO SE/SH, MO SE/DH). In addition, the rivalry rate analysis for the central-control condition is reported separately as well as part of the rivalry rate for the grouped percept. For the MO DE/SH and the MO DE/DH conditions (i.e., conducive to ungrouped percept), the rivalry rate analysis is irrelevant because our measure could not capture the switching rate within the unsynchronized percept (i.e., the rate of switching from horizontal to vertical for each rivalry target) and therefore it reflects an artificially reduced rivalry rate.

**FIGURE 4:**
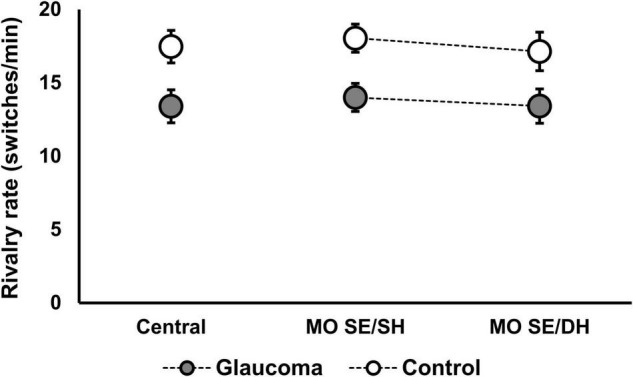
Rivalry rate for the grouped percept in the MO SE/SH and MO SE/DH conditions. Also shown, the rivalry rate for the central-control condition. Error bars are ± 1 SE.

The rivalry rate for the grouped percept was analyzed with a 2 (condition: MO SE/SH, MO SE/DH) × 2 (group: glaucoma, control) mixed factorial ANOVA. As the rivalry rate can be affected by age (Arani et al., 2019) and given that the glaucoma group was slightly — although not significantly — older, age adjustment was done by introducing age as a covariate in this analysis. The results revealed only that the group effect was significant, [*F*(1, 28) = 5.33, *p* = 0.029, partial η^2^ = 0.16]. Overall, the rivalry rate was significantly lower in the glaucoma group than in the control group, irrespective of condition; age did not affect these results. Further, we introduced the central-control condition in the model and analyzed the data with a 3 (condition: central-control, MO SE/SH, MO SE/DH) × 2 (group: glaucoma, control) mixed factorial ANOVA. For this analysis too, the results revealed only that the group effect was significant, [*F*(1, 28) = 5.98, *p* = 0.021, partial η^2^ = 0.18]. The results are shown in Table 3 and Figure 4. Separately for the central-control condition, an independent-sample *t*-test showed that the rivalry rate of the glaucoma group was significantly lower than that of the control group, [*t*(29) = 2.55, *p* = 0.016, Cohen’s *d* = 0.92]. The latter analysis is redundant, but we reported it only to emphasize that this specific result replicates our previous findings (Tarita-Nistor et al., 2020).

**TABLE 3:** Mean ± standard deviation of the rivalry rates in the central-control, MO SE/SH, and MO SE/DH conditions, for the two groups.

	Central	MO SE/SH	MO SE/DH
Glaucoma	13 ± 5	14 ± 4	13 ± 5
Control	17 ± 4	18 ± 4	17 ± 5

*Rivalry rates are measured in switches/min.*

### Rivalry Rate and Time Dominance of the Grouped Percept in the Left, Right, Upper, and Lower Locations

We examined whether the difference in the rivalry rate between the two groups was driven by a specific location. Rather than using the averaged data for the SH (i.e., left and right locations) and for the DH (i.e., upper and lower locations), we examined the rivalry rate for each location with a 4 (location: left, right, lower, upper) – 2 (group: glaucoma, control) mixed factorial ANOVA, using age as a covariate. As with the previous analysis, we found only that the group effect was significant, [*F*(1, 28) = 5.33, *p* = 0.029, partial η^2^ = 0.16], but the location and interaction effects were not significant. A similar analysis was conducted for time dominance of the exclusive grouped percept, but no significant differences were found.

### Epochs of Exclusive Dominance of the Grouped Percept

**FIGURE 5:**
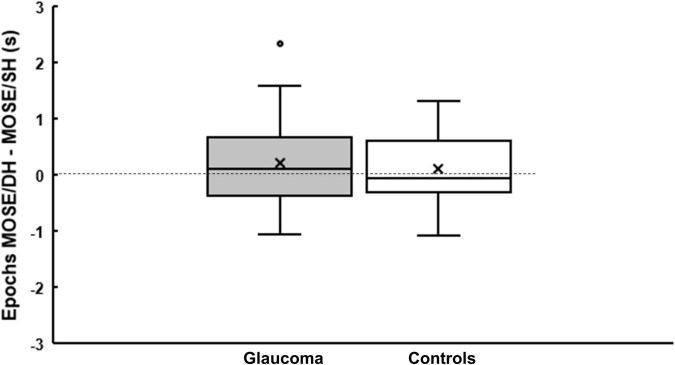
Epochs of the grouped percept dominance. Boxplots showing the difference in epochs of grouped percept dominance between MO SE/DH and MO SE/SH conditions for glaucoma and control group. The cross and the horizontal line inside the box represent the mean and the median, respectively. For the glaucoma group, data point shown as a small circle above the box plot’s whisker represents an outlier. The bottom and the top line of the box represent the first and third quartile, respectively.

In this analysis, we explored whether there was a difference in the epochs of the exclusive dominance of the grouped percept for within and between hemifields conditions. For this, we first calculated the epochs for the MO SE/SH and for the MO SE/DH conditions (as described in section “Data Analysis/Method”), and then computed the difference epochs: MO SE/DH—epochs MO SE/SH. This computation produced highly variable data, as illustrated by the boxplots in Figure 5. The epochs of exclusive dominance in the MO SE/SH condition were a median of 48-ms longer for the control group, but a median of 116-ms shorter for the glaucoma group when compared to those of the MO SE/DH condition. However, the Mann–Whitney *U*-test revealed a non-significant difference between the groups. This analysis was not appropriate for the other conditions where grouping did not occur, and where the rivalry rates were irrelevant.

### Relationships With Clinical Measures

For the glaucoma group, we examined whether there were any correlations between the rivalry rates for the grouped percept and monocular clinical measures averaged for the two eyes (i.e., visual field’s MD, RNFL thickness, and vertical cup-to-disc ratio). No significant relationships were found. We further examined whether there were any relationships between the rivalry rates and the absolute difference between the monocular clinical measures (i.e., the absolute value of the difference between clinical measures of the right and left eye). No meaningful relationships were found. We also examined the relationships between the rivalry rates for the grouped percept and binocular acuity as well as stereoacuity for both groups and found no evidence that any important relationships exist.

## Discussion

In this study, we examined perceptual grouping during the binocular rivalry in patients with mild glaucoma who otherwise had normal visual acuity and no or minimal visual field defects. The main findings are as follows: (1) For the glaucoma and control group, the grouping was strongest when the identical rivalry targets were presented to the same eye, irrespective of the hemifield; (2) the time dominance for the grouped as well as for the ungrouped percept were similar for both groups; (3) the rivalry rates were significantly lower in the glaucoma group than in the control group across all relevant conditions (i.e., MO SE/SH, and MO SE/DH); and (4) the epochs of exclusive dominance of the grouped percept in the within (i.e., MO SE/SH) and between hemifields (i.e., MO SE/DH) conditions had different patterns for the two groups. Taken together, these results support the view that the binocular rivalry processes are disrupted in early glaucoma (Tarita-Nistor et al., 2019, 2020), but whether the perceptual grouping is affected is inconclusive.

When two dissimilar images are presented dicoptically to the two eyes, the visual system cannot integrate them into one stable percept; rather, the two images compete for visual awareness, such as one dominates for a few moments and then the other, in a continuous cycle. This phenomenon is known as the binocular rivalry and it has been used as a tool to gain insights into the dynamics of the visual system and the cortical processing in healthy and in clinical populations. An interesting property of the binocular rivalry is that spatially separated rivalry targets with identical features tend to dominate at the same time; in other words, the two spatially separated targets group together during the rivalry. It has been suggested that the grouping is mediated by the interactions between the lateral connections of the cortical hypercolumns in the visual cortex (Alais and Blake, 1999), and therefore the weaker neural connectivity would affect grouping. Interestingly, grouping during the binocular rivalry is facilitated only under specific viewing conditions. It has been shown that the eye of origin of the two rivalry stimuli with similar features is the strongest cue for the grouping in healthy controls (Stuit et al., 2011, 2014). That is, grouping depends primarily on the eye of origin of the image, such that the strongest grouping happens when the identical rivalry targets are presented to the same eye (i.e., Figures 1A,B). This effect is very weak when the identical rivalry targets are presented to different eyes (i.e., Figures 1C,D). The current study replicates these results as follows: For both patients and controls, we found that the grouping occurred mainly in the conditions where the identical rivalry targets were presented to the same eye, irrespective of the hemifield (i.e., MO SE/SH and MO SE/DH conditions) while negligible grouping occurred when the identical targets where presented to different eyes (i.e., MO DE/SH and MO DE/DH conditions).

Although there was no difference in the time dominance of the grouped percept between the two groups, this measure may not be the most sensitive to detect changes in neural dynamics during the rivalry in mild glaucoma. Using a classical rivalry paradigm, we have previously shown that the patients and controls have a similar time of exclusive percept dominance, but the important differences were found in the rivalry rate: In conditions involving inter-hemispheric transfer of visual information (i.e., similar to the present central-control condition), the rivalry rate of the glaucoma group was significanlty lower than that of the control group (Tarita-Nistor et al., 2019, 2020). These findings have been replicated recently using stimuli of different size, orientation, and spatal frequency (João et al., 2021). Indeed, the rivalry rate has been used as the measure of choice to detect changes between healthy and clinical populations who otherwise have normal visual function. The rivalry rate depends on the balance of the exitatory/inhibitory interactions of populations of neurons, which may be determined by the glutamate/Gamma-Aminobutyric Acid (GABA) neurotransmitter balance; any dysregulation especially in the GABA levels may result in impairments in the rivalry rate (Laing and Chow, 2002; van Loon et al., 2013; Robertson et al., 2016). It has been shown that in people with pathological conditions with an abnormal pattern of neural excitatory/inhibitory interactions, such as autism spectrum disorder, bipolar disorder, schizophrenia, and major depression disorder, the rivalry rate is lower than that of the healthy controls (Robertson et al., 2013; Said et al., 2013; Jia et al., 2015). However, there is no convincing evidence that patients with glaucoma have a glutamate/GABAergic imbalance; rather, evidence exists for a widespread degeneration in neural structures across the whole brain (Gupta et al., 2006; Garaci et al., 2009; Hernowo et al., 2011; Zhang et al., 2012; Williams et al., 2013), and therefore it is more likely that impairments in the rivalry rate are due to neurodegenerative processes in these patients.

In this study, we replicated our previous report that patients with mild glaucoma have a lower rivalry rate than controls in conditions involving inter-hemispheric transfer of visual information (Tarita-Nistor et al., 2020). That is, for the central-control control condition (single rivalry targets) rivalry rate was lower in the glaucoma than in the control group. In addition, we found that the rivalry rate of the grouped percept was significantly lower in glaucoma than in control group, irrespective of the hemifield (i.e., MO SE/SH and MO SE/DH conditions). One question to consider is whether this overall decline in the rivalry rate is due to weaker connectivity between neighboring rivalry zones or is caused by impaired neural dynamics that affect the rivalry processes in glaucoma. Our data suggest that for the MO SE/SH condition (same hemifield) a lower the rivalry rate of the grouped percept may indicate weak grouping processes rather than diminished the rivalry dynamics. This is because we have been previously shown that in glaucoma, the rivalry rate of classical stimuli (single rivalry targets) is normal in the same hemifield conditions and therefore the current deficits observed in the MO SE/SH condition cannot be explained by impairments in the rivalry dynamics (Tarita-Nistor et al., 2020). We acknowledge, however, that the stimulus eccentricity, size, and spatial frequency were different in this study than in our previous work, but these factors are unlikely to account entirely for the present results. Interestingly, we expected an additive impairment caused by inter-hemispheric transfer dysfunction and weak grouping that should have resulted in a rivalry rate of the glaucoma group in the MO SE/DH condition (between hemifields) to be lower than that in the MO SE/SH condition (same hemifield) and also lower than that of the controls. Our hypothesis was only partially confirmed, and more research will be needed to untangle why the rivalry rates of the grouped percept were similar for the same (SH) and different hemifields (DH) for the glaucoma group. Therefore, we refrain from concluding that perceptual grouping is affected in these patients based only on these findings.

**FIGURE 6:**
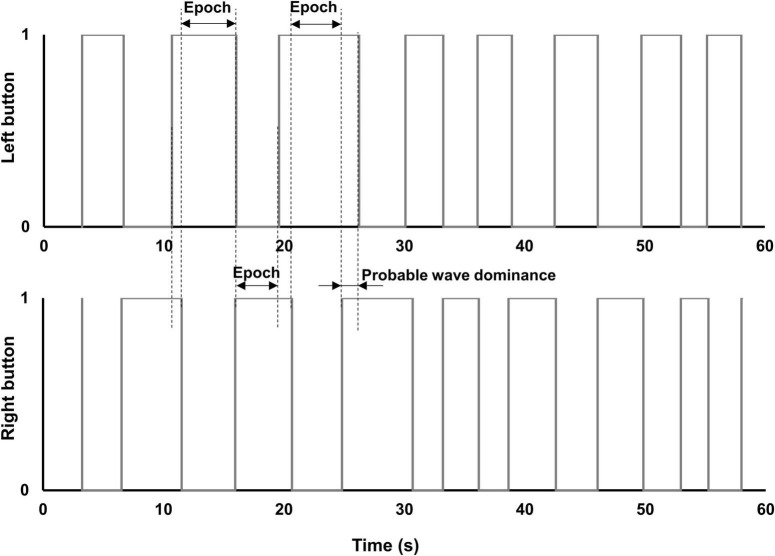
Diagrams of button-response box responses. Diagrams of epochs of exclusive dominance plotted from actual data produced by a patient with early glaucoma, in one of the MO SE/DH conditions. The small overlap of the right and left buttons in the “on” position (i.e., value “1”) indicates the change in percept dominance (i.e., probable wave dominance). This overlap was excluded from the epoch’s duration calculation.

To further investigate the differences in perceptual grouping during the binocular rivalry in the same (SH) and different hemifields (DH) for the two groups, we subtracted the epochs of exclusive dominance in the MO SE/SH condition from those in the MO SE/DH condition. This analysis was pertinent only for these two conditions, where grouping occurred and the rivalry rates were relevant. We found that the epochs of exclusive dominance in the MO SE/SH condition were a median of 48-ms longer for the control group, but interestingly, a median of 116-ms shorter for the glaucoma group when compared to those of the MO SE/DH condition. During rivalry, the percept dominance changes in a wave-like fashion and — in an ingenious experiment (Wilson et al., 2001) — it has been reported that in healthy controls the dominance wave propagation is about 173-ms longer in between than within hemifield conditions, likely due to a time penalty for propagation through the long callosal fibers. While in the current study, we did not expressly measure the dominance wave propagation, the probable waves were excluded from the epochs’ length calculation as shown in the logic timing diagrams, as shown in Figure 6, plotted from the data obtained with the button-response box. That is, longer waves would make the epochs shorter and shorter waves would make the epochs longer. For the control group, these results imply that the wave dominance of the grouped percept propagates faster in the same (SH) than in different hemifields (DH), and this agrees with predictions by Stuit et al. (2011). However, the opposite was true for the glaucoma group; this finding is counterintuitive but consistent with our previous study in which we directly measured the time of dominance wave propagation within and between hemifields (Tarita-Nistor et al., 2019). A faster dominance wave propagation in conditions involving inter-hemispheric transfer has been also found in patients with mild traumatic brain injury whose long axons in the neural bundle connecting the two hemispheres are most prone to damage (Spiegel et al., 2015). The consistency of the findings reported by the current study with past research is notable given the high variability of the data (Spiegel et al., 2015; Tarita-Nistor et al., 2019), although further research is warranted.

Neuroimaging studies have shown extensive changes in the gray and the white matter of the glaucomatous brain, affecting the whole primary visual pathways, visual association areas, the corpus callosum, as well as more distal areas, such as the frontoparietal cortex, cerebellar cortex, and hippocampi (Gupta et al., 2006; Garaci et al., 2009; Hernowo et al., 2011; Zhang et al., 2012; Williams et al., 2013; Yu et al., 2013; Frezzotti et al., 2014; Jiang et al., 2017; Nucci et al., 2020). These changes have been observed in the initial stages of the disease, with a more profound neurodegeneration as glaucoma progresses (Williams et al., 2013; Frezzotti et al., 2014). Using behavioral studies, we probed the functional integrity of specific neural structures and found impairments that otherwise could not be detected with standard clinical measures. The consistent effects we reported in studies involving vection (Tarita-Nistor et al., 2014; Brin et al., 2019), binocular rivalry (Tarita-Nistor et al., 2019, 2020), and now the perceptual grouping during the binocular rivalry also indicate neurodegenerative processes in early glaucoma. However, a clear link between degeneration in neural structures and these functional changes is still to be demonstrated and therefore future studies should include both imaging and behavioral measures to confirm that these functional impairments are triggered by neurodegeneration in early glaucoma.

In such a study, a replay of transition condition should be included as a control condition. This is a condition in which the same stimulus is presented to both eyes at any time, but the stimulus changes in a manner that simulates the visual perception during binocular rivalry, such as one stimulus is viewed first, then it transitions into the rivalrous stimulus, in a continuous cycle. The replay paradigm is an excellent tool used as a control condition in the brain imaging studies to help differentiate between the neural activation arising from the genuine binocular rivalry and the non-specific activation (Tong and Engel, 2001; Knapen et al., 2011; Roy et al., 2017). When used in pure behavioral studies, such as ours, a replay condition can reveal whether the clinical group has similar response characteristics as the control group. Although we did not use a replay of transition as a control condition in our current study, we have no reason to suspect that the two groups differ in response characteristics, given that the inclusion criteria required both the patients and the control subjects to be generally healthy, with no history of neurological diseases, cognitive impairment, or diabetes. Also, the two groups had the same time dominance results in all conditions, suggesting that the patients understood the task well. Moreover, it has been shown that the rivalry rates—the most relevant outcome reported in our study—are not seriously affected by the reaction time (Blake et al., 1992).

We acknowledge some limitations of this study. First, the eye movements were not monitored during this experiment (Hirasawa et al., 2018). We enrolled only patients with early glaucoma, with a normal visual acuity and with no visual field defects and excluded those with unreliable visual fields whose fixation losses exceeded 20%. Keeping a steady gaze on the fixation target during the binocular rivalry was essential and we provided constant reminders to the participants to do so. The changes in fixational stability secondary to glaucoma have been reported in those with mild-to-moderate visual field defects, but stronger evidence for fixation stability impairment in patients with early glaucoma, such as those included in this study is needed. For example, it has been reported that fixation stability of patients with glaucoma (i.e., mixed group of early and moderate stage) is worse than that of controls, but only on specific measures, such as the proportion of fixational eye position points within 2° diameter area (Shi et al., 2013) or when using the sequential Euclidian distance method for quantifying fixation (Montesano et al., 2018). When fixation stability was quantified as the proportion of fixational eye position points within 4°-diameter area (Shi et al., 2013) or with the bivariate contour ellipse area (Montesano et al., 2018), the differences between glaucoma and control groups were no longer significant. Second, blinks were also not monitored during the experiment. Patients with glaucoma can experience ocular surface disturbances more often than healthy control due to topical medication. However, we are not aware of any reports showing that patients with early glaucoma have a different pattern or frequency of blinks than controls (Gisler et al., 2015; Cho et al., 2018). We examined our unpublished preliminary eye movements data recorded from the patients with early glaucoma with no or minimal visual field defect included in a different study and found no evidence that these patients made a significantly larger number of blinks than controls. Finally, due to the restrictions imposed by the pandemic, we were not able to collect visual field and OCT data from the control group. All healthy controls confirmed that they had had a relatively recent ophthalmic examination with no findings of visual problems. If control participants had any eye pathologies, then it is likely that the rivalry rate would have been diminished for this group too; yet, we found a higher rivalry rate in this group compared to glaucoma group. Nevertheless, we acknowledge that all these issues are pertinent to the results presented in this study and should be addressed in future research.

## Conclusion

In conclusion, this study found abnormalities in the binocular rivalry in patients with mild glaucoma on a perceptual grouping during the rivalry task. However, evidence for impairments in perceptual grouping *per se* could be inferred only indirectly, and therefore we refrain from drawing this conclusion. These deficits may have implications for higher levels of visual processing, such as object recognition and scene segmentation, but this remains to be confirmed in future research.

## Data Availability Statement

The raw data supporting the conclusions of this article will be made available by the authors, upon request.

## Ethics Statement

The studies involving human participants were reviewed and approved by the University Health Network’s Research Ethics Board and the research was conducted in accordance with the tenets of the declaration of Helsinki. The patients/participants provided their written informed consent to participate in this study.

## Author Contributions

GI managed the project, participated in study design, performed patient recruitment, collected and interpreted the data, provided clinical expertise, and was a major contributor in writing the manuscript. GT provided resources, secured funding, participated in study design, interpreted data, provided clinical expertise, and was a contributor in writing the manuscript. YB provided resources, secured funding, participated in study design, interpreted data and was a contributor in writing the manuscript. LT-N conceptualized the study, provided resources, secured funding, analyzed and interpreted the data and was a major contributor in writing the manuscript. All authors contributed to the article and approved the submitted version.

## Conflict of Interest

The authors declare that the research was conducted in the absence of any commercial or financial relationships that could be construed as a potential conflict of interest.

## Publisher’s Note

All claims expressed in this article are solely those of the authors and do not necessarily represent those of their affiliated organizations, or those of the publisher, the editors and the reviewers. Any product that may be evaluated in this article, or claim that may be made by its manufacturer, is not guaranteed or endorsed by the publisher.
